# Fluorescent TAP as a Platform for Virus-Induced Degradation of the Antigenic Peptide Transporter

**DOI:** 10.3390/cells8121590

**Published:** 2019-12-07

**Authors:** Magda Wąchalska, Małgorzata Graul, Patrique Praest, Rutger D. Luteijn, Aleksandra W. Babnis, Emmanuel J. H. J. Wiertz, Krystyna Bieńkowska-Szewczyk, Andrea D. Lipińska

**Affiliations:** 1 Laboratory of Virus Molecular Biology, Intercollegiate Faculty of Biotechnology, University of Gdańsk, Abrahama 58, 80–307 Gdańsk, Poland; magda.wachalska@phdstud.ug.edu.pl (M.W.); malgorzata.graul@biotech.ug.edu.pl (M.G.); aleksandra.babnis@gmail.com (A.W.B.); krystyna.bienkowska-szewczyk@biotech.ug.edu.pl (K.B.-S.); 2 Department of Medical Microbiology, University Medical Center Utrecht, Heidelberglaan 100, 3584CX Utrecht, The Netherlands; P.Praest-2@umcutrecht.nl (P.P.); rdluteijn@berkeley.edu (R.D.L.); e.wiertz@umcutrecht.nl (E.J.H.J.W.)

**Keywords:** TAP-GFP, fluorescent TAP platform, antigen presentation, MHC I, immune evasion, BoHV-1 UL49.5

## Abstract

Transporter associated with antigen processing (TAP), a key player in the major histocompatibility complex class I-restricted antigen presentation, makes an attractive target for viruses that aim to escape the immune system. Mechanisms of TAP inhibition vary among virus species. Bovine herpesvirus 1 (BoHV-1) is unique in its ability to target TAP for proteasomal degradation following conformational arrest by the UL49.5 gene product. The exact mechanism of TAP removal still requires elucidation. For this purpose, a TAP-GFP (green fluorescent protein) fusion protein is instrumental, yet GFP-tagging may affect UL49.5-induced degradation. Therefore, we constructed a series of TAP-GFP variants using various linkers to obtain an optimal cellular fluorescent TAP platform. Mel JuSo (MJS) cells with CRISPR/Cas9 TAP1 or TAP2 knockouts were reconstituted with TAP-GFP constructs. Our results point towards a critical role of GFP localization on fluorescent properties of the fusion proteins and, in concert with the type of a linker, on the susceptibility to virally-induced inhibition and degradation. The fluorescent TAP platform was also used to re-evaluate TAP stability in the presence of other known viral TAP inhibitors, among which only UL49.5 was able to reduce TAP levels. Finally, we provide evidence that BoHV-1 UL49.5-induced TAP removal is p97-dependent, which indicates its degradation via endoplasmic reticulum-associated degradation (ERAD).

## 1. Introduction

The co-existence of a host and a virus depends on a subtle balance between the pathogen replication and the host immune response. Virus-derived peptides, originating mainly from the proteasomal degradation, are presented by the major histocompatibility complex class I (MHC I) molecules, leading to the recognition of an infected cell by cytotoxic CD8^+^ T lymphocytes (CTLs) (reviewed in [1]). The transporter associated with antigen processing (TAP) plays a pivotal role in MHC I-restricted antigen presentation, which makes it an attractive target for viruses that aim to escape the immune system.

TAP is a heterodimer belonging to the ATP-binding cassette (ABC) family transporters. It consists of two subunits, TAP1 (ABCB2) and TAP2 (ABCB3) [2]. The core of each subunit is formed by an N-terminally-located transmembrane domain (TMD), composed of six transmembrane helices (TMs), responsible for peptide recognition and binding [3], and a highly conserved C-terminal nucleotide-binding domain (NDB), which can bind and hydrolyze ATP [4]. Acquiring both substrates, ATP and the peptide, occurs independently [3]. This induces conformational rearrangements within TAP, resulting in a switch from an inward-open to an outward-facing conformation and release of the peptide into the lumen of endoplasmic reticulum (ER). Afterward, ATP hydrolysis triggers the release of phosphate and restores the resting state of TAP [5]. The presence of core-flanking TMD0 domains (four TMs in TAP1 and three TMs in TAP2) at the N termini of TAP1/TAP2 is not necessary for peptide transport; however, it is crucial for assembly of the peptide-loading complex (PLC) and subsequent exposure of antigenic peptides to CTLs [6].

During co-evolution with their hosts, several herpesviruses and a single known (to date) poxvirus have specialized in TAP inhibition via diverse mechanisms (reviewed in [7]). Herpes simplex virus 1 and 2 (HSV-1 and HSV-2) encode the ICP47 protein, which competes for the peptide-binding site and, through its characteristic structure, tethers the TAP-ICP47 complex in an inward-facing conformation [8,9,10]. In contrast, the US6 protein of human cytomegalovirus (HCMV) [11,12,13] and the cowpox virus (CPXV) strain Brighton Red-encoded CPXV012 protein can inhibit ATP binding to NDBs while leaving peptide binding unaffected [14,15,16]. Mechanisms of TAP inhibition by herpesvirus UL49.5 protein family encoded by members of the *Varicellovirus* genus are still not fully understood and seem to differ in detail between virus species. Most of the UL49.5 orthologs inhibit conformational rearrangements within TAP [17]. Bovine herpesvirus 1 (BoHV-1) UL49.5 seems to be unique in its ability to target bovine, human, and murine TAP for proteasomal degradation following the conformational arrest [7,18,19]. Varicella-zoster virus (VZV)-encoded UL49.5 can bind TAP, yet it exhibits no inhibitory properties [20]. TAP degradation activity was also described for the murine gammaherpesvirus-68 MK3 protein [21] and the rodent herpesvirus Peru pK3 ortholog [22], which both carry a cytoplasmic RING (really interesting new gene) finger domain and can act towards the murine transporter. The recently described poxvirus molluscum contagiosum virus MC80 protein can destabilize human TAP; however, in contrast to BoHV-1 UL49.5, the transporter is not the primary target of the inhibitor [23].

Recently, fluorescent tags and gene fusion technology have become indispensable in a wide range of biochemical and cell biology applications, nevertheless in some circumstances designing a functional fluorescent fusion protein remains challenging. Numerous studies have shown that a choice of a linker may have a significant impact on proper folding, yield, and functionality of the fusion protein and its interaction with other proteins. Flexible linkers are usually applied to provide a certain degree of movement, while rigid linkers are preferable to separate two bioactive domains spatially [24].

To investigate the mechanism of TAP inhibition or removal, a TAP-GFP (green fluorescent protein) fusion protein was instrumental, yet GFP-tagging was observed to abolish the susceptibility of TAP to degradation induced by the BoHV-1-encoded UL49.5 [18]. Here, we report the construction of a series of full-length TAP1 and TAP2 variants carrying either N- or C-terminal GFP with different types of linkers and evaluate the impact of the TAP-GFP fusion design on their fluorescence and functionality, as well as susceptibility to virus-induced inhibition and degradation. Such a fluorescent TAP platform may constitute a platform to explain the molecular mechanism of UL49.5 activity and potentially contribute to better characterization of the transporter itself.

## 2. Materials and Methods

### 2.1. Cells and Viruses

Madin-Darby bovine kidney (MDBK) cells (ATCC, Manassas, VA, USA, CCL-22), human melanoma Mel JuSo (MJS) cells, MJS TAP1 CRISPR/Cas9 knock-out (TAP1 KO), MJS TAP2 CRISPR/Cas9 knock-out (TAP2 KO) [25], and U937 (ATCC, CRL-1593) were cultured in RPMI 1640 (Corning, Corning, NY, USA) supplemented with 10% fetal bovine serum (FBS, Thermo Scientific (Thermo Scientific, Waltham, MA, USA)) and Antibiotic Antimycotic Solution (Thermo Scientific). Lenti-X HEK293T and GP2-293 cells (both from Takara/Clontech, Kusatsu, Japan) used for lentivirus and retrovirus production, respectively, were cultured in Iscove’s modified Dulbecco’s medium (IMDM, Lonza, Basel, Switzerland) supplemented as above. HEK293T (ATCC, CRL-3216) cells were cultured in Dulbecco’s modified Eagle’s medium (DMEM, high glucose, Lonza) supplemented as above. BoHV-1 field strain Lam (Institute for Animal Health and Science, Lelystad, The Netherlands) was propagated and titrated on MDBK cells.

### 2.2. DNA Constructs

All TAP constructs were cloned in lentiviral vectors downstream of an EF1α promoter.

**Figure 1:**
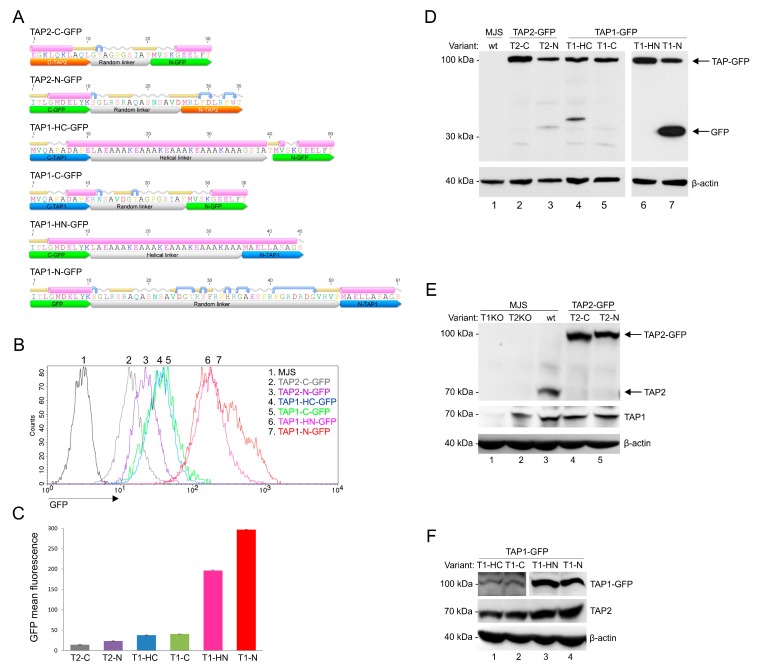
Construction and characterization of fluorescent transporter associated with antigen processing (TAP)-green fluorescent protein (GFP) variants. Mel JuSo (MJS) cells with CRISPR/Cas9 TAP1 or TAP2 knockouts (T1KO, T2KO) were stably reconstituted with fluorescent TAP1 or TAP2 constructs using lentivirus vectors and cell sorting. (**A**) Schematic representation of TAP-GFP constructs. Secondary structures of linkers flanked by ten amino acid residues of fused proteins were determined by the Geneious software; α-helices are depicted in pink, coiled regions in gray, β-strands in yellow, and turns in blue. (**B**) Representative histograms of TAP-GFP fluorescence intensity. (**C**) Comparative TAP-GFP analysis by flow cytometry. The mean fluorescence intensity of three independent measurements is represented as bars with standard deviations. The statistical significance was assessed by *t*-test; *p* ≤ 0.001. (**D**–**F**) Expression of TAP-GFP variants in stable cell lines was determined by SDS-PAGE and immunoblotting using: (**D**) anti-GFP monoclonal antibody (Mab) (**E**) anti-TAP2 MAb (**F**) anti-TAP1 MAb. β-actin was used as a loading control. Abbreviations: T2-C: TAP2-C-GFP (TAP2 with the C-terminal GFP, random linker); T2-N: TAP2-N-GFP (TAP2 with the N-terminal GFP, random linker); T1-C: TAP1-C-GFP (TAP1 with the C-terminal GFP, random linker); T1-HC: TAP1-HC-GFP (TAP1 with the C-terminal GFP, helical linker); T1-N: TAP1-N-GFP (TAP1 with the N-terminal GFP, random linker); T1-HN: TAP1-HN-GFP (TAP1 with the N-terminal GFP, helical linker).

For unmodified (wild-type, wt) TAP1 or TAP2 reconstitution, dual promoter lentiviral vectors described in [25] (pPuroR-GFP-TAP1 and pZeoR-mAmetrine-TAP2) were used. mAmetrine and marker GFP genes were removed from these vectors. Fragments of TAP1 and TAP2 sequences were ordered as synthetic genes designed for cloning in pEGFP-N3 or pEGFP-C1 (Takara/Clontech). For TAP1-N-GFP (TAP1 with the N-terminal GFP, random linker), TAP1-C-GFP (TAP1 with the C-terminal GFP, random linker), TAP2-N-GFP (TAP2 with the N-terminal GFP, random linker), and TAP2-C-GFP (TAP2 with the C-terminal GFP, random linker), fusion genes were re-cloned in the original lentiviral vectors. The amino acid sequences of random linkers resulting from the cloning procedure are depicted in Figure 1A. Fragments coding for TAP1 with helical linker sequences were ordered as synthetic genes designed for cloning in pEGFP-N3 or pEGFP-C1. TAP1-HN-GFP (TAP1 with the N-terminal GFP, helical linker) or TAP1-HC-GFP (TAP1 with the C-terminal GFP, helical linker) were re-cloned in the lentiviral vector pCDH-EF1α-MCS-(PGK-Puro) (System Biosciences, Palo Alto, CA, USA).

Genes coding for viral TAP inhibitors were cloned in retroviral vectors downstream of a retroviral promoter. The BoHV-1 UL49.5 gene was cloned from pLZRS-BoHV-1 UL49.5-IRES-GFP [18] in BamHI-EcoRI sites of pLZRS-IRES-ΔNGFR [26]. The VZV UL49.5 gene was amplified from the pLZRS-VZV UL49.5-IRES-GFP vector [20] using KOD Hot Start DNA polymerase (Merck, Darmstadt, Germany) and the following primers: forward 5’-CGGGATCCCACCATGGGATCAATTACC-3’ and reverse 5’-CCGGAATTCTTACCACGTGCTGCGTAATAC-3’. The PCR product was verified by DNA sequencing and introduced into BamHI and EcoRI sites of the pBABEpuro vector [27]. Synthetic genes encoding: myc-tagged HSV-1 ICP47 (Gene ID: 2703441), myc-tagged HCMV US6 (Gene ID: 3077555), or the FLAG-N-CPXV012 (Gene ID: 1485887) variantlacking six N-terminal amino acid residues [28] were introduced into BamHI and EcoRI restriction sites of pBABEpuro.

### 2.3. Retroviral and Lentiviral Transduction

For the production of recombinant lentiviruses, third-generation packaging vectors based on the pRSV-Rev and pCgpV plasmids (Cell Biolabs, San Diego, CA, USA), the obtained lentiviral expression vectors, and pCMV-VSV-G (Cell Biolabs) for pseudotyping were co-transfected into Lenti-X HEK293T cells using CalPhos mammalian transfection kit (Takara/Clontech). For recombinant retroviruses, a transfer plasmid (pBABEpuro-based or pLZRS-IRES-ΔNGFR-based) and pCMV-VSV-G were co-transfected into GP2-293 packaging cells as above. Twenty-four hours after transfection the medium was refreshed; for lentiviruses it was supplemented with 1 mM sodium butyrate (Sigma-Aldrich, Saint Louis, MO, USA). Virus-containing supernatants were collected after 48 h, concentrated with PEGit (System Biosciences), and used for transduction in the presence of 0.01 mg mL^−1^ polybrene (Sigma-Aldrich). MJS cells with TAP1 or TAP2 knock-outs were stably reconstituted with the wt or fluorescent TAP1 or TAP2 constructs using lentivirus vectors and cell-sorting for GFP- and MHC I-positive cells. The cells were subsequently transduced with a retrovirus coding for BoHV-1 UL49.5 and sorted for nerve growth receptor (NGFR)-positive cells or with a retrovirus coding for HSV-1 ICP47, HCMV US6, VZV UL49.5, or CPXV012, and selected with puromycin (2 µg mL^−1^) (Sigma-Aldrich). 

### 2.4. Plasmid Transfection

HEK293T cells were transfected with plasmids encoding fluorescent TAP variants using JetPRIME (Polyplus-transfection, Illkirch, France) according to the manufacturer’s protocol and analyzed after 24 h by flow cytometry.

### 2.5. Generation of A TAP1/TAP2 Double Knock-Out U937 Cells for Reconstitution with Fluorescent TAP Variants

U937 TAP1/TAP2 KO cells were generated with a strategy described for MJS TAP1/TAP2 KO in [25] Briefly, U937 cells were transfected with pSico-CRISPR-PuroR containing the TAP2-targeting crRNA sequence 5’-GGAAGAAGAAGGCGGCAACG-3’. The cells were selected with puromycin (4 µg mL^−1^), and cloned by limiting dilution. Individual clones were analyzed by flow cytometry to identify clones with low cell surface MHC I expression, followed by immunoblotting and DNA sequencing of the genomic target site. A clone lacking TAP2 was subsequently transfected with a pSicoR-CRISPR-PuroR vector containing the TAP1-targeting crRNA sequence 5’-GGGGTCCTCAGGGCAACGGT-3’. After selection with puromycin and cell cloning, the clones were analyzed for TAP1 expression by immunoblotting and DNA sequencing of the genomic target site. Genomic DNA sequence analysis revealed a 16-bp deletion around the TAP2 gRNA target site and multiple short deletions altering the whole TAP1 gene sequence downstream of the target site. A monoclonal cell line lacking TAP1 and TAP2 was used for reconstitution with a combination of unmodified and fluorescent TAP-encoding sequences delivered by lentivirus vectors. Reconstituted U937 cells were sorted for GFP and high MHC I expression. The cells were subsequently transduced with the BoHV-1 UL49.5-encoding retrovirus and sorted for NGFR.

### 2.6. Antibodies

Antibodies used for immunoblotting: mouse anti-TAP1 monoclonal antibody MAb 143.5 (kindly provided by R. Tampé, Institute of Biochemistry, The Johann Wolfgang Goethe University, Frankfurt, Germany); mouse anti-TAP2 MAb 435.3 (a kind gift from P. van Endert, INSERM U25, Institute Necker, Paris, France); rabbit anti-TAP1 (Enzo Life Sciences, Farmingdale, NY, USA); rat anti-GFP 3H9 (Chromotek, Planegg, Germany); mouse anti-myc tag 9B11 (Cell Signaling, Danvers, MA, USA); rabbit anti-β-actin (Novus Biologicals, Centennial, CO, USA); rabbit anti-β-catenin (Santa Cruz Biotechnology, Dallas, TX, USA); rabbit antibodies (H11) against a synthetic peptide derived from the N-terminal domain of BoHV-1 UL49.5 [26] and mouse anti-OctA (FLAG) G-8 (Santa Cruz Biotechnology); and mouse anti-HC10 [19] and rabbit anti-ERp57 H-220 (Santa Cruz Biotechnology). Probes used for immunofluorescence: Alexa 633-conjugated concanavalin A (ConA) (Thermo Scientific). Antibodies used for flow cytometry: mouse anti-MHC I W6/32 (Novus Biologicals); mouse anti-NGFR (Sigma-Aldrich); and Alexa 633-conjugated goat anti-mouse IgG (Thermo Scientific).

### 2.7. Flow Cytometry

Cell surface expression of MHC I was determined by indirect immunofluorescence using primary anti-MHC I antibodies (1:1000) and secondary antibodies (1:1000), all in phosphate buffered saline (PBS) buffer containing 1% bovine serum albumin and 0.02% sodium azide. For cell sorting, anti-NGFR antibodies (1:1000) and secondary antibodies were used. Cells were analyzed using a FACS Calibur flow cytometer (Becton Dickinson, Franklin Lakes, NJ, USA) and CellQuest software (version 5.2.1, Becton Dickinson)); for cell sorting, the sorting option of FACS Calibur was applied.

### 2.8. Immunoblotting and Immunoprecipitation

For immunoblotting, the cells were lysed in Cell Lytic M buffer (Sigma-Aldrich); for immunoprecipitation, the cells were lysed in a buffer containing 1% digitonin (Merck), 50 mM Tris-HCl, pH 7.5, 5 mM MgCl_2_, and 150 mM NaCl. The buffers were supplemented with the cOmplete mini protease inhibitor cocktail (Roche, Basel, Switzerland). Cell lysates were analyzed by SDS-PAGE and immunoblotting as previously described [26] or incubated with GFP-Trap (Chromotek) according to the manufacturer’s protocol to isolate protein complexes.

### 2.9. Peptide Transport Assay

The peptide transport assay was performed as described before [26]. Briefly, the cells were permeabilized with 2 IU mL^−1^ of Streptolysin O (Sigma-Aldrich) at 37 °C for 15 min. The cells (2 × 10^6^ cells/sample) were subsequently incubated with 600 pmol of the fluorescein-conjugated synthetic peptide CVNKTERAY (JPT Peptide Technologies, Berlin, Germany) in the presence or absence of ATP (10 mM final concentration) at 37 °C for 10 min. Peptide translocation was terminated by adding 1 mL of ice-cold lysis buffer containing 1% Triton X-100. After 20 min of lysis, cell debris was removed by centrifugation, and supernatants were collected and incubated with 100 µL of concanavalin A (ConA)-Sepharose (Sigma-Aldrich) at 4 °C for 1 h to isolate the glycosylated peptides. After extensive washing of the beads, the peptides were eluted with elution buffer (500 mM mannopyranoside, 10 mM EDTA, 50 mM Tris-HCl, pH 8.0) by rigorous shaking for 1 h at 25 °C. Eluted peptides were separated from ConA by centrifugation at 12,000× *g*. The fluorescence intensity was measured using a fluorescence plate reader (EnVision, PerkinElmer, Waltham, MA, USA) with excitation and emission wavelengths of 485 nm and 530 nm, respectively.

### 2.10. Immunofluorescence

MJS cells were grown on microcover glass, fixed with 4% paraformaldehyde in PBS, permeabilized with 0.2% Triton X-100 in PBS, and stained with Alexa 633-ConA (1:1000), prepared in PBS containing 1% bovine serum albumin (Sigma-Aldrich). GFP booster (1:100, Chromotek) was used for MJS-TAP2-C-GFP to enhance the green fluorescence. The blue signal was electronically converted into the red during the analysis of images using Leica TCS SP8X confocal laser scanning microscope (Leica, Wetzlar, Germany).

## 3. Results

### 3.1. Construction and Characterization of Fluorescent TAP-GFP Variants

In order to develop a universal fluorescent platform for virus-induced TAP degradation, we constructed six versions of TAP-GFP fusion: four with two types of linkers, a random linker or a helical one, placed at the N- or C-terminus of TAP1, and two with a random linker at the N- or C-terminus of TAP2 (Figure 1A). A number of studies regarding fusion protein linker design have suggested that the most important features of a proper linker are its hydrophilicity and flexibility [24]. The random linkers used to join TAP and GFP have resulted from the cloning procedure into one of the pEGFP plasmid series. The analysis of their amino acid sequence revealed they were unstructured; thus, they should be more flexible. In some cases, flexible linkers may result in undesired interactions or interference between the fusion partners. In such cases, rigid linkers are preferable to separate two independently active domains spatially. An example of a rigid linker is the α helix-forming peptide AEAAAKEAAAKEAAAKA, stabilized by salt bridges between glutamate and lysine residues [29]. The distance between two separated domains can be regulated by changing the number of EAAAK motif repetitions. By using fluorescence resonance energy transfer (FRET) measurement, the helical linker with four repetitions of this motif has been demonstrated as the most efficient in separating two fluorescent proteins [30]. Therefore, this linker was selected for our studies to generate N- and C-terminal fusion of TAP1 and GFP. To introduce fluorescent TAP-GFP into human melanoma MJS cells, we first generated, by using CRISPR/Cas9-based technology, TAP1 or TAP2 knock-outs (KO). This enabled stable reconstitution of the cells with a fluorescent TAP1 or TAP2 using lentiviral vectors. The cells were subsequently sorted based on GFP to high purity (>98%). MJS cells have been shown to be permissive for BoHV-1 infection and are widely used in the research on modulation of antigen presentation by viruses [31,32,33].

First, we analyzed the GFP fluorescence intensity of our constructs (Figure 1B,C). Flow cytometry analysis revealed that N-terminal fusions of GFP to TAP1 exhibited the highest fluorescence, followed by TAP1 C-terminal fusion constructs. The type of linker seemed to have no crucial impact on the fluorescence intensity, although, for TAP1-N-GFP, a population of brighter green fluorescent cells could be observed when compared to TAP1-HN-GFP. TAP2 constructs exposed the lowest fluorescence, with a similar tendency of N-terminal fusion outperforming the C-terminal one. In addition, for TAP1-N-GFP, we could observe a more heterogeneous distribution of GFP fluorescence than for the other variants.

During the transient expression of fluorescent constructs in plasmid-transfected HEK293T cells, we could observe a similar range of GFP fluorescence, which indicates that differences in GFP fluorescence depend on the properties of individual fusion proteins rather than result from random incorporation of a lentivirus into a host genome (Figure S1). TAP2-C-GFP was performing noticeably better in transfected HEK293T than in the stable MJS cell line, and this time TAP1-HN-GFP slightly outperformed TAP1-N-GFP.

Next, we characterized the expression of TAP-GFP constructs by SDS-PAGE and immunoblotting. TAP1 and TAP2 have a similar apparent molecular mass of approximately 75 kDa. The fusion to GFP should yield a single protein at 100 kDa. All constructs were detected in cell lysates with anti-GFP antibodies at the predicted molecular weight of 100 kDa; only for TAP1-N-GFP did we observe an additional 30-kDa band corresponding to, most probably, cleaved free GFP or cleaved TAP-GFP fragment (Figure 1D). Fluorescent TAP could also be detected with anti-TAP1 or anti-TAP2 antibodies (Figure 1E,F). The reconstitution of TAP1KO cells with a fluorescent variant of TAP1 resulted in increased stability of endogenous TAP2 as it could be detected in higher amounts than in TAP1KO cells (compare lane 1 in Figure 1E with lanes 1–4 in Figure 1F). This is in agreement with a previous study reporting that unlike TAP1, TAP2 is unstable when expressed without the other half of the transporter [34]. The higher sensitivity of TAP2 can most probably explain why no band corresponding to TAP2 could be identified in MJS TAP1KO cells (Figure 1E, lane 1), while TAP1 could be easily detected in MJS TAP2KO cells (Figure 1E, lane 2).

### 3.2. TAP-GFP Localizes in the ER and Forms a Functional Transporter

**Figure 2:**
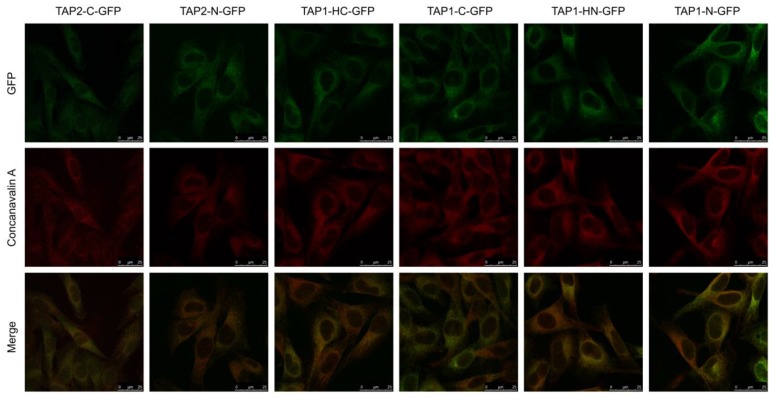
TAP-GFP variants demonstrate endoplasmic reticulum (ER) localization. MJS cells reconstituted with TAP-GFP variants were fixed, permeabilized, and stained with Alexa 633-conjugated concanavalin A (ER marker). Colocalization with GFP was analyzed using fluorescent confocal microscopy. For MJS TAP2-C-GFP, the GFP booster was used to enhance very weak green fluorescence.

TAP localizes in the cells predominantly in the ER [35]. To assess if the fluorescent TAP constructs acquired their proper localization, we stained the cells with Alexa 633-conjugated concanavalin A (ConA), an ER-cis Golgi marker, and analyzed fluorescence distribution by confocal laser scanning microscopy (Figure 2). In all cell lines, TAP-GFP (green) localized predominantly in the ER (red), as it co-localized with the ER marker (yellow). No granular localization patterns that would indicate TAP aggregates could be visualized.

**Figure 3:**
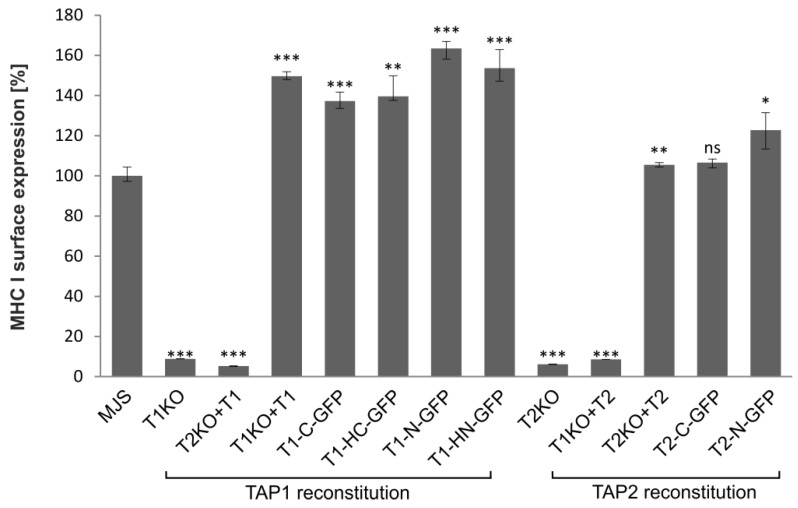
TAP-GFP forms a functional transporter. MJS cells with CRISPR/Cas9 TAP1 or TAP2 knockouts (T1KO or T2KO) were stably reconstituted with wild-type or fluorescent TAP1 or TAP2 variants. Surface expression of major histocompatibility complex class I (MHC I) was assessed by flow cytometry using specific antibodies (W6/32). MHC I expression on MJS cells with TAP reconstitution is presented as the percentage of MHC I mean fluorescence intensity on MJS cells (set as 100%). The analysis was performed in triplicates. The statistical significance of differences between MHC I on MJS cell with TAP reconstitution and MJS wild-type (wt) cells was estimated by *t*-test; *** *p* ≤ 0.001, ** *p* ≤ 0.01, * *p* ≤ 0.05, ns: not significant.

Based on the currently available evidence, a lack of at least one fully functional TAP subunit results in a suppressed peptide translocation and production of empty or suboptimally loaded unstable MHC I molecules, mostly retained in the ER [36]. GFP fusion could potentially affect TAP structure and activity. Therefore, the functionality of fluorescent TAP constructs was tested by measuring cell surface expression of MHC I by flow cytometry (Figure 3). As expected, MJS cells with TAP1KO or TAP2KO exhibited strongly reduced levels of MHC I. To have a proper control for testing the effect of fluorescent tagging on TAP performance, especially during overexpression of a TAP subunit, we generated control cell lines reconstituted with unmodified TAP subunits (MJS TAP1KO + TAP1 and MJS TAP2KO + TAP2). Overexpression of individual TAP subunits might result in the formation of their homodimers, affecting the interpretation of our further experiments [19,37]. Therefore, as an additional control, we transduced TAPKO cells with vectors enabling overexpression of the existing half-transporter (MJS TAP1KO + TAP2 and MJS TAP2KO + TAP1). As expected, MHC I surface levels were reduced in those cells to a similar extent as in TAP1KO or TAP2KO cells. On the other hand, reconstitution of the missing TAP subunit with its fluorescent variant rescued surface MHC I to levels slightly higher than on parental (“wild-type”) MJS cells, but similar to the ones observed on the cells reconstituted with the non-fluorescent TAP.

### 3.3. The Sensitivity of TAP-GFP Variant to UL49.5-Mediated MHC I Downregulation and TAP Degradation

**Figure 4:**
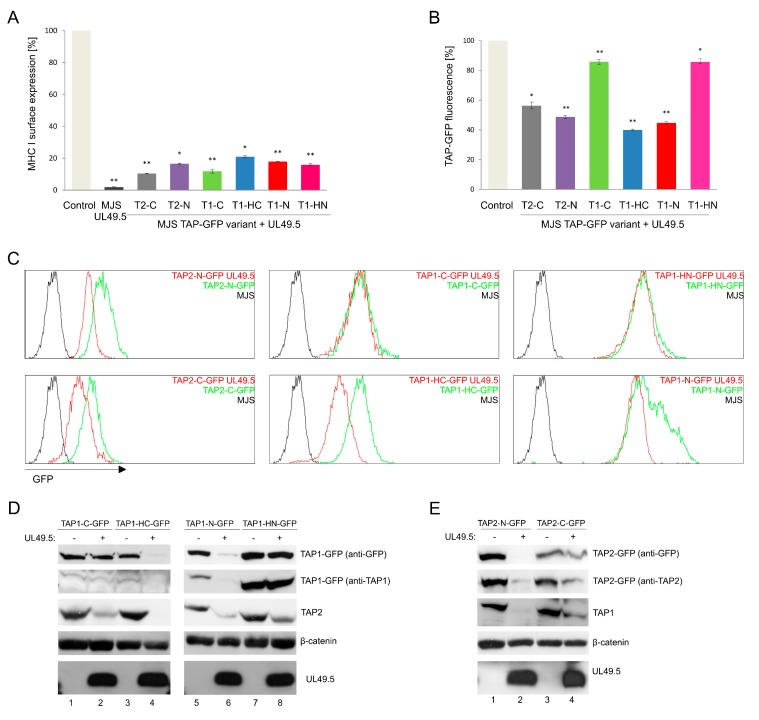
TAP-GFP variants differ in their sensitivity to UL49.5-mediated inhibition and degradation. MJS cells with fluorescent TAP1 or TAP2 variants were transduced with a retrovirus encoding bovine herpesvirus 1 (BoHV-1) UL49.5. (**A**) Surface expression of MHC I was assessed by flow cytometry using specific antibodies (W6/32). MHC I expression is presented as the percentage of mean fluorescence intensity; fluorescence of parental cells without UL49.5 was set as 100%. The analysis was performed in triplicates. The statistical significance of differences between MJS TAP-GFP and MJS TAP-GFP UL49.5 cell lines was estimated by *t*-test; ** *p* ≤ 0.001 * *p* ≤ 0.005. (**B**) GFP mean fluorescence intensity is presented as the percentage of GFP fluorescence of parental cells (set as 100%). The analysis was performed in triplicates. The statistical significance of differences between MJS TAP-GFP and MJS TAP-GFP UL49.5 cell lines was estimated by *t*-test; ** *p* ≤ 0.001 * *p* ≤ 0.005. (**C**) The effect of UL49.5 on GFP level in MJS TAP-GFP cells was assessed by flow cytometry. (**D**,**E**) Degradation of TAP-GFP variants in the presence of BoHV-1 UL49.5 in stable cell lines was determined by SDS-PAGE and immunoblotting using: anti-GFP, anti-TAP1, anti-TAP2, or anti-UL49.5 antibodies. β-catenin was used as a loading control.

To examine the sensitivity of fluorescent TAP to BoHV-1-encoded UL49.5-mediated inhibition, UL49.5 was introduced to stable cell lines with TAP-GFP variants using a retrovirus vector. The cells were subsequently sorted for high purity based on the expression of truncated NGFR as a marker, and analyzed by flow cytometry for surface expression of MHC I (Figure 4A). According to the obtained data, all fluorescent transporters were prone to UL49.5-induced inhibition, which was illustrated as the downregulation of MHC I, to a similar extent for all the tested variants. 

Next, we analyzed the susceptibility of TAP-GFP variants to UL49.5-triggered degradation (Figure 4B,C). Flow cytometry analysis revealed a reduction of GFP mean fluorescence intensity by approximately 50% in the cells co-expressing UL49.5 with TAP1-N-GFP, TAP1-HC-GFP, and both TAP2 variants, in comparison to the control cells expressing the fluorescent transporter without UL49.5. There were no significant changes in GFP fluorescence observed in the cells expressing TAP1-C-GFP or TAP1-HN-GFP with UL49.5.

Susceptibility of TAP1-HC-GFP and TAP2-N-GFP constructs to UL49.5-dependent TAP inhibition and degradation was also confirmed in a reconstituted U937 cell line (Figure S2), where downregulation of surface MHC I, as well as a decrease of mean GFP fluorescence to the similar extent as in MJS cells, could be denoted. 

To verify if changes in GFP fluorescence correspond with the decreased TAP protein level, immunoblotting analysis of cell lysates was performed (Figure 4D,E). A similar level of BoHV-1 UL49.5 protein could be observed in all cell lines. Decreased amounts of TAP1-N-GFP, TAP1-HC-GFP, and both TAP2-GFP fusion proteins could be detected in the presence of UL49.5, with the use of both anti-GFP and specific anti-TAP antibodies. Of note, in the case of non-degradable fluorescent TAP1 constructs (TAP1-HN-GFP and TAP1-C-GFP), the level of endogenous TAP2 was decreased (compare TAP2 in Figure 4D in lanes 1–2 and 5–6), what may suggest partial degradation of the untagged TAP subunit only. Degradation in TAP1-HC-GFP and TAP2-N-GFP cell lines was observed for both, exogenous fluorescent and endogenous TAP subunits. TAP1-N-GFP and TAP2-C-GFP characterized with only partial degradation.

### 3.4. Interaction of TAP-GFP with UL49.5 and the Peptide-Loading Complex

**Figure 5:**
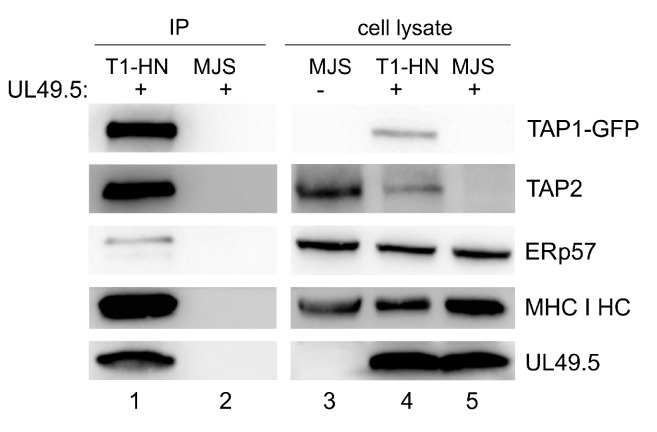
TAP1-GFP interacts with UL49.5 and the peptide-loading complex. TAP1-HN-GFP (T1-HN) was immunoprecipitated by GFP-Trap from lysates of MJS cells expressing TAP1-HN-GFP and UL49.5 or wt MJS with UL49.5 only. Co-precipitating proteins were analyzed by SDS-PAGE and immunoblotting using antibodies against GFP, TAP2, ERp57, MHC I HC, and UL49.5. Right panel: cell lysates were loaded on SDS-PAGE directly and analyzed by immunoblotting.

To assess the interaction of a non-degradable TAP with BoHV-1-encoded UL49.5 and selected components of PLC, we chose the TAP1-HN-GFP construct as demonstrating high expression. Among proteins co-immunoprecipitating with the fluorescent transporter (using GFP-Trap), we could identify UL49.5 and endogenous TAP2, as well as the known components of PLC: MHC I heavy chain and ERp57 (Figure 5). These results highlight the fact that UL49.5 is still capable of interacting with the non-degradable TAP1-HN-GFP. Unspecific binding of UL49.5 was excluded by immunoprecipitation from MJS wt cell lysate (with unmodified TAP) expressing UL49.5.

### 3.5. Application of the TAP2-N-GFP Variant as a Platform to Study BoHV-1 UL49.5 Activity in Virus-Infected Cells

In the more detailed studies on our fluorescent TAP platform, we focused on a single TAP-GFP variant, selecting TAP2-N-GFP. TAP1-N-GFP was excluded based on the presence of a free form of GFP. The constructs resistant to UL49.5-triggered degradation were eliminated as well, as was TAP2-C-GFP due to its very week, nearly undetectable basic green fluorescence and only partial degradation in the presence of UL49.5.

**Figure 6:**
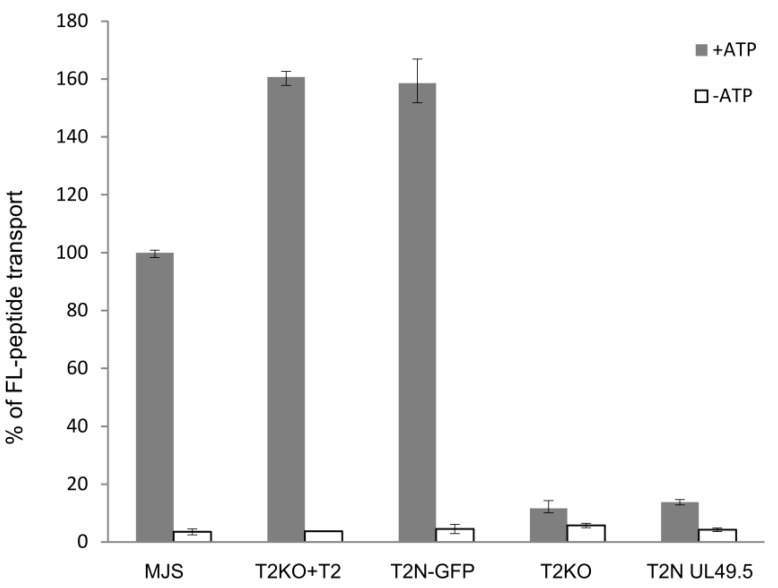
UL49.5-induced inhibition of peptide transport. MJS cells with CRISPR/Cas9 TAP2 knockout (T2KO) were stably reconstituted with wild-type TAP2 (T2KO+T2) or fluorescent TAP2 (T2N-GFP), subsequently transduced with a retrovirus encoding BoHV-1 UL49.5, and sorted (T2N UL49.5). Transport activity of TAP was analyzed using fluorescein-labeled peptide CVNKTERAY in the presence of ATP (gray bars) or EDTA (white bars). Peptide transport is expressed as a percentage of translocation, relative to the translocation observed in control MJS cells (set at 100%). The experiment was performed in triplicates. The statistical significance of differences between MJS controls and reconstituted or KO variants was estimated by *t*-test; for all the samples *p* ≤ 0.005.

First, we confirmed that UL49.5-mediated MHC I downregulation relies on the inhibition of antigenic peptide translocation. TAP transport assay was performed in TAP2-N-GFP MJS cells and TAP2-N-GFP cells expressing UL49.5 or (as controls) in MJS wt, MJS TAP2KO cells, and MJS TAP2KO cells reconstituted with a non-fluorescent TAP2. The assay was based on cytoplasm-to-ER translocation of fluorescein-conjugated substrate peptides, in the presence of ATP or EDTA, as a passive diffusion control (Figure 6). Reconstitution with fluorescent TAP restored peptide transport when compared to the parental TAP2KO cells to a level of almost 50% higher than for MJS wt cells. This might result from high expression of exogenous TAP2 as a very similar transport activity was denoted for the non-fluorescent TAP2 reconstitution. The presence of UL49.5 inhibited peptide translocation to the level of TAP2KO cells.

**Figure 7:**
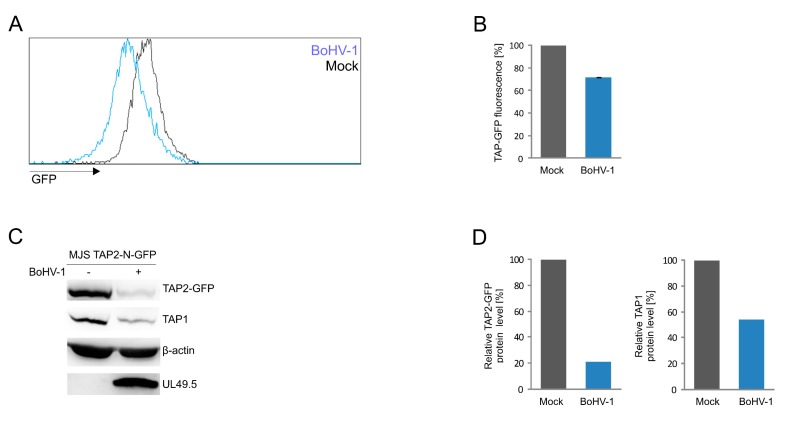
BoHV-1 infection results in TAP2-GFP degradation. MJS TAP2-N-GFP cells were infected with BoHV-1 at a multiplicity of infection (moi) = 10. Twenty-four hours post-infection, cells were collected and analyzed. (**A**) TAP2-GFP fluorescence was assessed by flow cytometry; histograms from a representative analysis are shown, and (**B**) depicted as the percentage of fluorescence in mock-infected MJS TAP2-N-GFP cells (set as 100%). The analysis was performed in triplicates. The statistical significance was assessed by *t*-test; *p* ≤ 0.0005 (**C**) TAP2-GFP degradation was determined by SDS-PAGE and immunoblotting using anti-GFP, anti-TAP1, or anti-UL49.5 antibodies; β-actin was used as a loading control. (**D**) The relative amount of TAP2-GFP detected by immunoblotting was normalized to β-actin.

Finally, to test whether we can apply our fluorescent TAP platform to quantify the TAP level during virus infection, we infected MJS TAP2-N-GFP cells with BoHV-1 and analyzed TAP-derived GFP fluorescence by flow cytometry (Figure 7A,B). TAP2-N-GFP and endogenous TAP1 levels were assessed in infected cell lysates by immunoblotting (Figure 7C,D). The viral infection resulted in a 30% decrease of mean GFP fluorescence intensity, while approximately 70% and 50% reduction of TAP2-N-GFP and TAP1 protein levels, respectively, could be demonstrated by immunoblotting.

### 3.6. Only UL49.5 among Different Viral TAP Inhibitors Can Induce Human TAP Degradation

**Figure 8:**
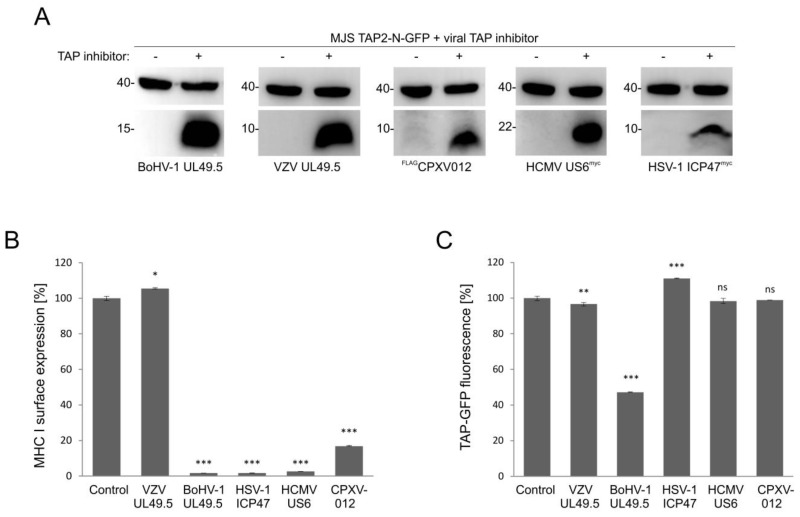
BoHV-1 UL49.5-mediated TAP degradation is unique among viral inhibitors of human TAP. MJS TAP2-N-GFP cells were transduced with a retrovirus encoding BoHV-1 UL49.5, varicella-zoster virus (VZV) UL49.5, human cytomegalovirus (HCMV) US6, herpes simplex virus 1 (HSV-1) ICP47, and cowpox virus CPXV012 and selected with puromycin. (**A**) The presence of viral TAP inhibitors was confirmed by SDS-PAGE and immunoblotting using anti-β, anti-BHV-1 UL49.5, anti-VZV UL49.5, anti-c-myc for HSV-1 ICP47, and HCMV US6 or anti-FLAG antibodies for CPXV012; β-actin (upper panels) was used as a loading control. Size markers are in kDa. (**B**) Surface expression of MHC I was assessed by flow cytometry using specific antibodies (W6/32). MHC I expression is presented as the percentage of MHC I on MJS TAP2-N-GFP cells (set as 100%). The analysis was performed in triplicates. The statistical significance of differences between MJS TAP2-N-GFP cells and cells with viral inhibitor was assessed by *t*-test; *** *p* ≤ 0.001 * *p* ≤ 0.05. (**C**) The mean fluorescence intensity of GFP was analyzed by flow cytometry and presented as the percentage of GFP fluorescence of MJS TAP2-N-GFP cells (set as 100%). The analysis was performed in triplicates. The statistical significance of differences between MJS TAP2-N-GFP cells and cells with a viral inhibitor was assessed by *t*-test; *** *p* ≤ 0.001 ** *p* ≤ 0.01 ns: not significant.

The effect of viral TAP inhibitors on TAP stability was reported, usually, in separate studies. TAP levels were analyzed in those reports by immunoblotting. To compare the sensitivity of fluorescent TAP to different viral inhibitors, we selected several representatives with distinct modes of action. Those were: competition for peptide (HSV-1 ICP47) or ATP (HCMV US6 and CPXV012) binding, as well as conformational arrest and degradation (BoHV-1 UL49.5) or a TAP-binding protein with no activity towards the transporter (VZV-encodedUL49.5). MJS TAP2-N-GFP cells were transduced with a retrovirus vector encoding a viral TAP inhibitor and subsequently selected to high purity. The presence of inhibitors was determined by immunoblotting (Figure 8A). Downregulation of MHC I surface expression could be observed during flow cytometry analysis, as expected, for all but the VZV-encoded protein (Figure 8B). Mean GFP fluorescence intensity measurement illustrated that BoHV-1 UL49.5 was unique in causing TAP-GFP degradation. ICP47 even seemed to slightly stabilize TAP-GFP (Figure 8C). 

### 3.7. UL49.5-Induced TAP-GFP Degradation Is p97-Dependent

**Figure 9:**
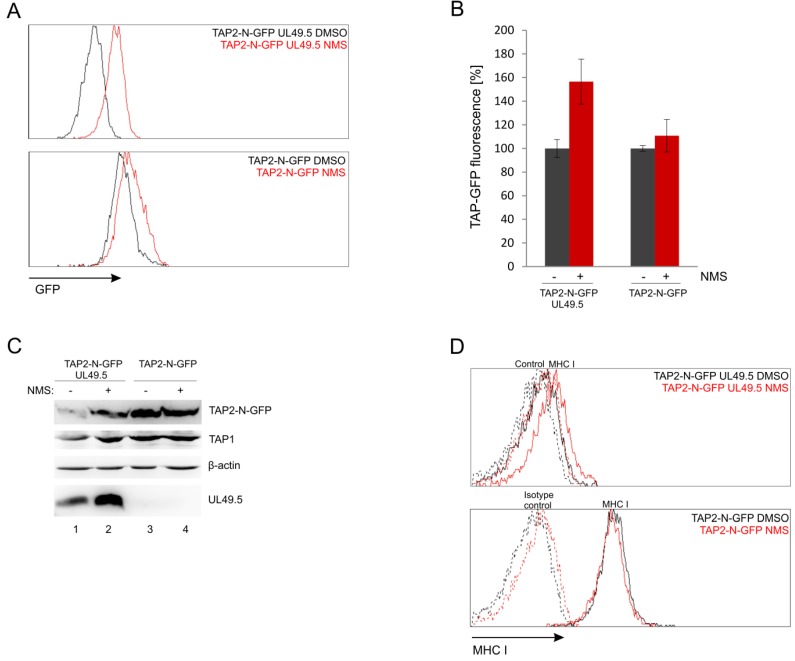
UL49.5 induced TAP-GFP degradation is p97-dependent. MJS TAP2-N-GFP UL49.5 cells were treated with p97 inhibitor NMS-873 (NMS) at 2 µM concentration for 24 hours. (**A**) Flow cytometry analysis of GFP fluorescence in NMS-treated and control (DMSO-treated) cells. (**B**) Relative GFP fluorescence in NMS-treated cells calculated as a percentage of GFP fluorescence in the control cells. The analysis was performed in triplicates. (**C**) The level of TAP-GFP in the presence of p97 inhibitor was determined by SDS-PAGE and immunoblotting using anti-GFP, anti-TAP1 or anti-UL49.5 antibodies; β-actin was used as a loading control. (**D**) Surface expression of MHC I assessed by flow cytometry using specific antibodies (W6/32).

The fluorescent TAP platform can be potentially applied to search for cellular proteins involved in the activity of BoHV-1 UL49.5. According to previous studies, UL49.5-induced TAP degradation is proteasome-dependent [18]. Since TAP is an ER-resident protein, it is presumed to be removed via one of the endoplasmic reticulum-associated degradation (ERAD) pathways. To verify this hypothesis, we used NMS-873 (NMS), an allosteric inhibitor of p97/VCP (valosin-containing protein) a major AAA ATPase belonging to ERAD [38]. MJS TAP2-N-GFP cells with or without UL49.5 were treated with a 2 µM concentration of NMS for 24 h and analyzed by flow cytometry. Inhibition of p97 drastically rescued the mean fluorescence intensity of TAP2 in the presence of UL49.5 to 170% of mean fluorescence intensity in cells treated with DMSO, while in TAP2-N-GFP cells without UL49.5 we could observe only minimal increase to 115% (Figure 9A,B). Compatible results were obtained during immunoblotting analysis of cell lysates (Figure 9C, compare TAP2-GFP in lanes 1–2 and 3–4). Interestingly, inhibition of p97 seemed to stabilize also the expression of UL49.5 (Figure 9C, lanes 1–2). However, apparently, this did not result in a more pronounced TAP-GFP degradation when p97 was blocked.

Next, by using flow cytometry, we determined how inhibition of p97 influences MHC I cell surface expression in the presence (and also absence) of UL49.5. NMS-873-treated cells had only slightly improved surface MHC I levels (Figure 9D). This effect was reminiscent of MHC I levels in the presence of some UL49.5 point mutants, which lost the ability to induce TAP degradation, like, for instance, mutants in the C-terminal RGRG motif [32].

## 4. Discussion

Quantitative studies on protein stability and degradation may require proper tools and platforms that grant full functionality of the protein of interest and, at the same time, assess the protein levels accurately. Here, we tested the application potential of various full-length TAP to GFP fusion constructs for the studies on TAP stability in the presence of four inhibiting proteins encoded by viruses, to obtain the most suitable fluorescent TAP platform. All the tested TAP-GFP variants were functional. Nevertheless, our results point toward a critical role of GFP localization on fluorescence intensity of the tagged transporter, which in concert with the type a linker used to separate TAP and GFP may regulate its susceptibility to virally induced degradation. By using this platform, we also provide evidence that BoHV-1 UL49.5-induced TAP degradation is p97-dependent. 

In a study on HSV-1 ICP47, a truncated fluorescent TAP complex (the so-called 6+6 transmembrane TAP core C-terminally fused to mVenus or mCerulean) was used to determine the effect of the viral protein on TAP thermostability [39]. However, for the studies on UL49.5, which was our primary protein of interest, the full-length TAP should constitute a better platform, as N-terminal TMD0s are required for maximum efficiency of UL49.5 binding and inhibition [19]. Full-length fluorescent TAP has been successfully used in several basic studies, some of which were to elucidate the association of H2L^d^ molecules with the TAP complex [40], follow lateral mobility of TAP in living cells [41], or illustrate its cellular distribution [42,43]. In those reports, the addition of a relatively large GFP tag to a much larger multiple membrane-spanning partner protein was tolerated to grant proper localization and functionality of the transporter. However, when exploited in a study on varicellovirus immune evasion, GFP-tagged TAP (C-terminal fusion using a random linker) failed to be degraded by BoHV-1 UL49.5, contrary to non-fluorescent wt TAP [18]. Since BoHV-1-encoded UL45.9 has been, so far, the only known viral inhibitor which can cause human TAP degradation apart from its inhibition, further investigation into this mechanism seemed very intriguing, and for this purpose, construction of fluorescent TAP was instrumental. 

Designing an optimal fluorescent TAP construct was hampered by the lack of complete structural information about TAP-UL49.5 interaction, and thus, it required an experimental evaluation of different TAP-GFP variants. The latest structural study on BoHV-1 UL49.5 revealed its 3D structure, while subsequent molecular docking experiments proposed three different possible orientations of TAP-UL49.5 complex in which UL49.5 was suggested to interact simultaneously with both TAP subunits [44]. However, these models were predicted based on the structure of ICP47-arrested TAP conformation [10], and therefore the actual UL45.9-TAP binding model needs to be further confirmed. 

Fluorescence analysis of constructed TAP-GFP variants in stable MJS cell lines provides evidence that the tag location, rather than the type of a linker used to separate TAP and GFP, has a pivotal impact on fluorescence intensity. N-terminal fusions generally granted stronger fluorescence (Figure 1B,C and Figure S1). It is worth mentioning here that both ends of TAP1 are present in the cytoplasm, while TAP2 incorporates its C terminus in the cytoplasm, and the N terminus localizes to the ER lumen [45,46]. Together with the fact that fluorescence of both TAP1 fusions was more intense than of TAP2, our results lead to speculations that it is the structure of both TMD0 and C-terminal NBDs that determines the fluorescent potential of the tagged constructs. For some constructs, especially for TAP1-N-GFP, we could observe additional protein products reacting with GFP-specific antibodies (Figure 1D), which might correspond to cleaved GFP and could also, most probably, explain higher and heterogeneous GFP signal of this construct observed by flow cytometry. The reason for the presence of free GFP in the case of TAP1-N-GFP is not fully understood. The length of this linker exceeds the size of other tested linkers, so it has a higher chance of affecting the stability of the protein. Another explanation might be the presence of a sequence recognized by cellular proteases, but the ExPASy PeptideCutter software analysis (https://web.expasy.org/peptide_cutter) did not reveal any significant candidates. 

Fluorescent tagging did not affect the subcellular localization and function of the transporter, even upon overexpression of only one TAP subunit (Figure 2 and Figure 3). Both TAP1 and TAP2 lack an N-terminal signal sequence for ER targeting [47], and the exact ER-targeting or ER-retention signals have not been identified to date. This encouraged us to design N-terminal GFP fusions with no additional signaling sequences preceding the tag. The localization of our constructs resembles patterns previously described for other recombinant fluorescent TAP proteins [38,39,40,41]. Our results stay in line with the studies on truncated TAP1/TAP2 [43] or functional dissection of transmembrane regions of TAP [6], which have indicated that the transmembrane segments themselves determine ER-localization. It is interesting that even genetically separated TMD0 and the core domains of TAP1 and TAP2 were previously found in the ER (TMD0 additionally localizing to the ER-Golgi intermediate compartment (ERGIC)), when co-expressed [6]. 

Replacing endogenous TAP1 or TAP2 with TAP-GFP or the untagged subunit restored MHC I on a cell surface equally well and to a level higher than on MJS wt cells (especially in the case of TAP1 constructs). One possible explanation could be the stronger stabilization of endogenous TAP2 by overexpression of TAP1. This effect might be especially noticeable in MJS cells since many melanoma-derived lines have lower endogenous expression of TAP, normally limiting MHC I surface levels [48]. Transduction of MJS TAP1KO or TAP2KO cells with the endogenously present subunit of the transporter did not increase MHC I level, which stays in line with the current view that although TAP1 and TAP2 can form homodimers under certain conditions, they are not functional in antigen presentation [19,35].

One of the most important results of this work provides evidence that all TAP-GFP variants were susceptible to UL49.5-induced inhibition to a similar extent, as assessed by surface MHC I downregulation (Figure 4A). However, only some of them were prone to degradation (both TAP2 fusions, TAP1-N-GFP and TAP1-HC-GFP, Figure 4B,D,E). TAP1-C-GFP remained resistant to UL49.5 what stays in agreement with the previous report [18]. As an interpretation of these data, we can suggest that the helical linker, in contrast to the random one, located at the C terminus of TAP1, effectively separates TAP from GFP to enable undisturbed TAP-UL49.5 interaction, resulting eventually in TAP degradation. Alternatively, it may also permit better access to ERAD components. In the case of the fluorescent TAP2 subunit, the location of GFP, despite the presence of random linkers, did not affect degradation, which could arise from structural differences between the TAP subunits. The TAP2 construct with GFP located in the ER lumen (TAP2-N-GFP) manifested more prominent degradation than the one with GFP in the cytoplasm. An additional observation from this experiment demonstrates that even in the case of a non-degradable fluorescent TAP variant, the second untagged endogenous TAP subunit seems to be sensitive to UL49.5-induced degradation (Figure 4D,E). This, in our opinion, supports the idea of reduced access to ERAD components in TAP1-C-GFP, whereas the access of the second untagged destabilized subunit remains, in this case, undisturbed. It is still unsolved whether UL49.5 can bind single TAP subunits, and the current mechanism points out at the heterodimer as the primary target [19]. As in MJS cells with non-degradable TAP variants, we could observe very efficient MHC I reduction, and the PLC composition in those cells seemed to be intact (Figure 5), at least with regard to the interaction of TAP with ERp57 and MHC I; our data confirm the previous report by [18], demonstrating that abolished degradation does not exclude inhibition. It even seems that TAP degradation might be only an auxiliary event, a “finish-off” effect, in the mechanism of UL49.5 action.

For further studies, we selected and validated TAP2-N-GFP as the most promising variant. TAP transport assay performed on this cell line confirmed that changes in MHC I surface levels reflect TAP transport efficiency (Figure 6). TAP transport in reconstituted cell lines, either with wt or a fluorescent version of the TAP subunit, was higher than in wt MJS. Then we demonstrated that results obtained in a stable cell line model system reflect a situation that occurs upon BoHV-1 infection, which was illustrated as loss of GFP fluorescence observed by flow cytometry and reduction of protein level shown by immunoblotting (Figure 7).

A former pulse-chase experiment with the use of proteasome inhibitor postulated co-degradation of TAP with UL49.5 [18]. In line with this working model, our data show that inhibition of p97 increases levels of both TAP and UL49.5, and demonstrate for the first time that UL49.5-induced TAP degradation requires functional p97. Most of known ER-resident substrates of this ATPase, which retrotranslocates proteins back to the cytoplasm, are ubiquitinated and targeted for proteasomal degradation [38], indicating that UL49.5 mediated TAP degradation occurs via ERAD.

Finally, the TAP2-N-GFP construct was verified as a platform for different viral TAP inhibitors, representing distinct mechanisms of transport inhibition and, most probably, binding another TAP conformation [28,39,49]. BoHV-1, HSV-1, and HCMV-encoded proteins were capable of drastic reduction of surface MHC I; CPXV012 contributed to a slightly weaker but still significant downregulation of MHC I, whereas VZV UL49.5, as expected, did not cause any changes. In terms of degradation, only BoHV-1 UL49.5 was able to decrease TAP-GFP levels, while ICP47 seemed even to stabilize TAP, which is in accordance with its reported effect on TAP thermostability. We believe that the fluorescent TAP platform provides more quantitative data in this respect when compared to previous immunoblotting analyses, which generally are more technically error-prone. 

## 5. Conclusions

In this study, we were able to validate the application potential of fluorescent TAP as a platform for viral immune evasion studies. Our results indicate TAP-GFP variants susceptible to BoHV-1 UL49.5-induced degradation, demonstrate that this degradation is p97-dependent, and emphasize the importance of linker design in fusion protein construction. The fluorescent TAP platform can be now applied in further research on BoHV-1 UL49.5, for instance in the genome-wide search for cellular proteins responsible for UL49.5-induced degradation, where the fluorescent signal can be measured and indicate even small changes in TAP levels. TAP-GFP could be also exploited to identify the active motifs or amino acid residues of UL49.5 affecting TAP stability. The same platform with viral inhibitors can be applied, in a similar way as in the study by [28], to identify TAP conformation recognized by UL49.5.

## Supplementary Materials

The following are available online at https://www.mdpi.com/2073-4409/8/12/1590/s1. Figure S1: Comparison of GFP fluorescence of TAP-GFP variants in HEK293T cells; Figure S2: Susceptibility of TAP-GFP expressed in reconstituted U937 cells to UL49.5-mediated inhibition and degradation.
