# Supplementary material for: Fluorescent TAP as a Platform for Virus-Induced Degradation of the Antigenic Peptide Transporter
Source: Cells. 2019 Dec 7;8(12):1590. doi: 10.3390/cells8121590 (PMC6952996; doi:10.3390/cells8121590)
Supplement: Supplementary file 1 [file cells-08-01590-s001.pdf]

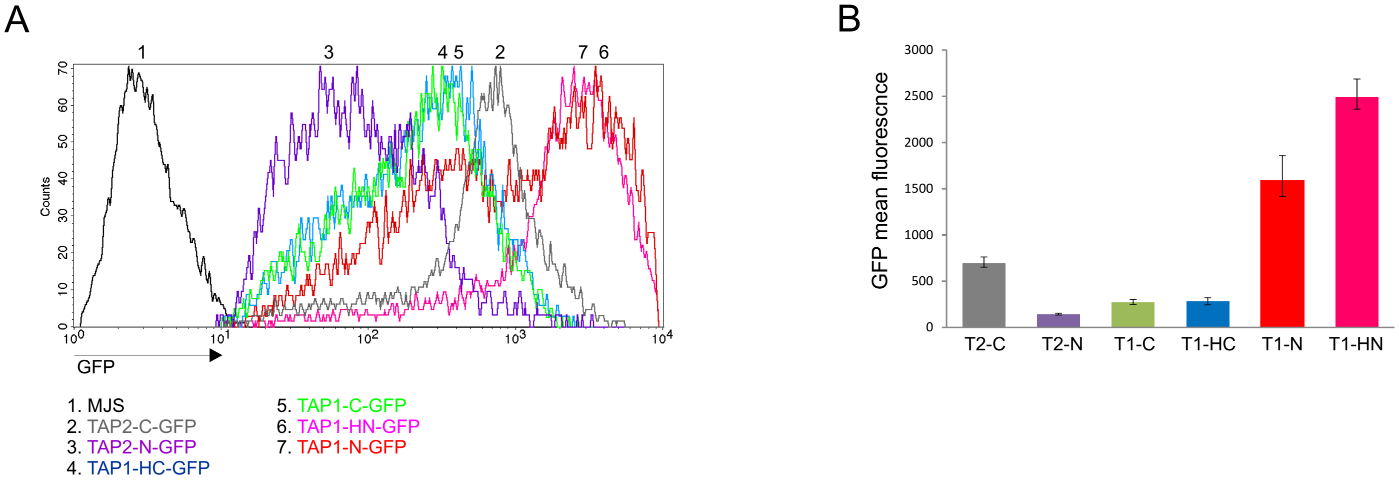

Supplementary Figure S1. Comparison of GFP fluorescence of TAP-GFP variants in HEK293T cells.
HEK293T cells were transfected with plasmids encoding different TAP-GFP variants. A. Fluorescence intensity was analyzed by flow cytometry. B. GFP mean fluorescence was analyzed by flow cytometry; the statistical significance was assessed by t-test; $p \leq 0{,}01$.

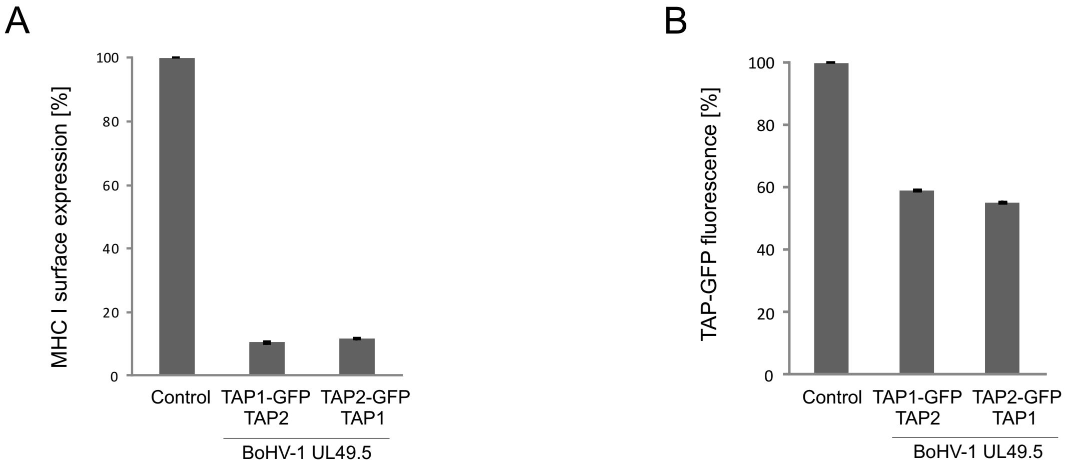

Supplementary Figure S2. Susceptibility of TAP-GFP expressed in reconstituted U937 cells to UL49.5-mediated inhibition and degradation.
U937 TAP1 and TAP2 KO cells reconstituted with a combination of a fluorescent and an unmodified TAP subunit were transduced with a retrovirus encoding BoHV-1 UL49.5. A. Surface expression of MHC I was assessed by flow cytometry using specific antibodies (W6/32). MHC I expression is presented as the percentage of mean fluorescence intensity; fluorescence of parental cells without UL49.5 was set as 100%. The analysis was performed in triplicates. The statistical significance of differences between U937 TAP-GFP and U937 TAP-GFP UL49.5 cell lines was estimated by t-test; $p \leq 0{,}001$. B. GFP mean fluorescence intensity is presented as the percentage of GFP fluorescence of parental cells (set as 100%). The analysis was performed in triplicates. The statistical significance of differences between U937 TAP-GFP and U937 TAP-GFP UL49.5 cell lines was estimated by t-test; $p \leq 0{,}001$. Abbreviations:TAP1-GFP – TAP1-HC-GFP; TAP2-GFP – TAP2-N-GFP.
